# MODEST: a web-based design tool for oligonucleotide-mediated genome engineering and recombineering

**DOI:** 10.1093/nar/gku428

**Published:** 2014-05-16

**Authors:** Mads T. Bonde, Michael S. Klausen, Mads V. Anderson, Annika I.N. Wallin, Harris H. Wang, Morten O.A. Sommer

**Affiliations:** 1 Novo Nordisk Foundation Center for Biosustainability, Technical University of Denmark, DK-2970 Hørsholm, Denmark; 2 Department of Systems Biology, Technical University of Denmark, DK-2800 Lyngby, Denmark; 3 Department of Systems Biology, Columbia University College of Physicians and Surgeons, NY 10032, USA

## Abstract

Recombineering and multiplex automated genome engineering (MAGE) offer the possibility to rapidly modify multiple genomic or plasmid sites at high efficiencies. This enables efficient creation of genetic variants including both single mutants with specifically targeted modifications as well as combinatorial cell libraries. Manual design of oligonucleotides for these approaches can be tedious, time-consuming, and may not be practical for larger projects targeting many genomic sites. At present, the change from a desired phenotype (e.g. altered expression of a specific protein) to a designed MAGE oligo, which confers the corresponding genetic change, is performed manually. To address these challenges, we have developed the MAGE Oligo Design Tool (MODEST). This web-based tool allows designing of MAGE oligos for (i) tuning translation rates by modifying the ribosomal binding site, (ii) generating translational gene knockouts and (iii) introducing other coding or non-coding mutations, including amino acid substitutions, insertions, deletions and point mutations. The tool automatically designs oligos based on desired genotypic or phenotypic changes defined by the user, which can be used for high efficiency recombineering and MAGE. MODEST is available for free and is open to all users at http://modest.biosustain.dtu.dk.

## INTRODUCTION

Recombination-mediated genetic engineering (recombineering) is readily applied for introduction of genetic variation in various organisms. Most often, longer inserts containing selectable markers were previously used to make one genomic change at a time. The use of short, single-stranded oligos has been shown to allow genomic modifications at high efficiencies in *Escherichia coli* (1–3) and *Saccharomyces cerevisiae* (4), thereby abolishing the need for a selectable marker (1). This allows rapid generation of mutants with specific changes and enables modification of many genomic sites at once, e.g., by multiplex automated genome engineering (MAGE) (1,5). These genome engineering methods are fast and efficient, requiring only standard laboratory equipment, for introducing simple mutations such as individual gene knockouts and amino acid substitutions. The creation of large cell libraries with targeted diversity can be achieved using many different oligos at once and performing several cycles of recombineering either manually or with automated approaches (1,4–5).

The experimental process is simple and involves (i) induction of the recombineering system, (ii) preparation of electrocompetent cells, (iii) addition of oligos and electroporation, and (iv) plating and identification of recombinant clones using multiplex allele specific colony (MASC) polymerase chain reaction (PCR) (1,5). However, designing oligos for recombineering and MAGE can be tedious, time-consuming, and often leads to sub-optimal oligos in terms of recombination efficiency. This can be a significant hindrance for implementation of multiplex genome engineering and makes larger scale projects, involving modifications of many targets, impractical or even impossible (e.g. for several thousand oligos).

**Figure 1.**
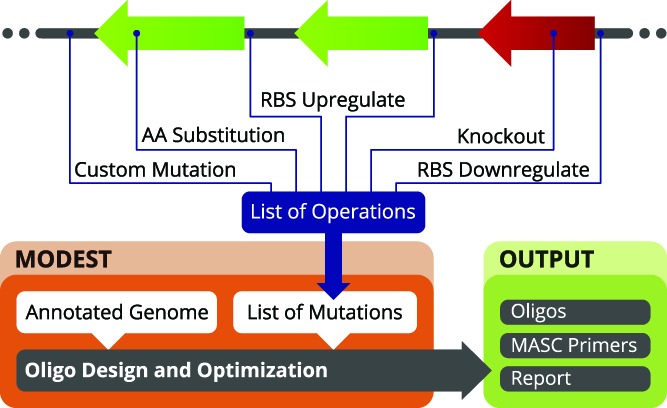
Concept flowchart of MODEST. The user selects or supplies an annotated genome along with a specification of the desired mutations, i.e. insertions, deletions, point mutations and amino acid substitutions, or phenotypic changes such as changes in the rate of protein translation and gene knockouts. MODEST processes this into a list of mutation objects, which are passed to an oligo design and optimization routine. This results in the design of MAGE oligos, MASC PCR primers, a report, and visualization of results.

In this study, we present MODEST, a web server for automatic design of optimized oligos, for recombineering and MAGE. The user can specify the desired mutations in a variety of input formats, and MODEST will design and optimize oligos, thereby introducing the desired genetic change (Figure 1). Target genomes are selected from a list, automatically loaded into the web server from NCBI by entering a genbank ID, or uploaded as a genbank file. The user can specify positions and desired mutations in drop-down menus and text fields, select specific genes, or provide a DNA sequence, including an indication of modifications. MAGE oligos are designed on the basis of the specified genetic change and are subsequently optimized. The optimization process involves shifting the location of the mutation within the oligo while evaluating the self-folding energy of the oligo. The oligo with the highest self-folding energy, where the mutations are at least 15 bp away from each end, is selected to avoid loss of the genetic change because of oligo processing by exonucleases (1,6–7).

Several modules are built on top of the oligo creation routine. The gene knockout module identifies putative mutations that should create a stable knockout of a gene by introducing several tandem stop codons. The translation rate module allows the user to specify a desired translation rate or set of translation rates, and MODEST will calculate mutations to achieve this with the least number of modifications. For this module, we developed a Monte Carlo simulation coupled with evaluation of translation rates based on a prediction algorithm previously developed (8). A visualization of the designed MAGE oligos and achieved expression level for each oligo versus the wild-type level is provided in the output.

A successful allelic replacement can be verified through MASC PCR and gel electrophoresis. MODEST automatically designs MASC PCR primers for PCR products of different lengths that can be used to identify mutant and wild-type colonies by employing different forward primers specific for the mutant and wild-type sequence, respectively (5).

Oligos created with MODEST are optimal for use with MAGE and recombineering methods where the methyl-directed mismatch repair (MMR) system is unfunctional, e.g., by using a MutS mutant strain (1) or a system where the MMR system can be temporarily knocked out (9). Sawitzke *et al.* (6) points out that the MMR system can be avoided by adding multiple specific changes in an oligo, and we are planning to develop an algorithm with this functionality in a future update.

## MATERIALS AND METHODS

### Specifying mutation targets

The input format for full organism sequences is an annotated GenBank file. The MODEST web server defaults to *E. coli* K12 MG1655. Other genomes can be used by either uploading a GenBank file or entering a GenBank sequence accession number for MODEST to automatically download. MODEST requires additional information on the target genome that is not available from the GenBank file, namely the replication origin and terminus, to design MAGE oligos targeting the lagging strand of DNA replication (6,10). This information can be supplied via the web interface or as a file upload. The parsing and automatic fetching of GenBank files is handled by the Biopython package (11). Bacterial genomes are fully supported at this time, but eukaryotic chromosomes and multiple origins are not. We plan to add this functionality in a future update.

The genomic changes are input as a white-space separated file, which can be dynamically created through the web interface, pasted onto the web interface, or uploaded. When a target genome is selected using the web server, the dynamic interface will automatically load the annotated genes and display them along with the available MODEST operations. When a gene or an operation is selected in the menu, relevant information such as gene sequence, documentation, and examples are loaded onto the sidebar.

### Oligo design and optimization

When a job is submitted, including specification of target genome and desired modifications, MODEST performs module-specific operations and returns a mutation object. The simplest available operation applies the mutation module, which accepts a desired nucleotide mutation, verifies it, and returns a mutation object. More complex modules include the gene knockout and translation rate modules. The internals of MODEST include a framework designed to rapidly expand and program new modules without changing the MODEST internals or the web server.

After a list of mutation objects is created, it is passed to the oligo creation and optimization routine. This routine extracts a length of nucleotides from the genomic sequence, upstream and downstream of the mutation target site, to obtain a full-length oligo based on user preference. Some studies have found 90 bp oligos to be optimal when cost is disregarded (1), while others have found shorter oligos of 60–70 bp to be equally efficient (6). The desired oligo length is a balance between targeting efficiency, cost of synthesis, and error rate of oligos (which is higher for longer oligos), and thus depends largely on individual preferences and future expanded studies. We therefore set the default oligo length for MODEST to 90 bp, but with a possibility to easily change it under the advanced options at the web server.

If necessary, the sequence is reverse complemented to target the lagging strand (3). To perform this operation, the location of the oligo is determined relative to the known origin and terminus of replication. Commonly used genomes with annotated or predicted origin and terminus of replication is available directly in MODEST and listed with details and corresponding methods for recombination with single-stranded oligos (12–15) in Table 1. MODEST can automatically detect annotated origin of replication as is the case for *E. coli* K12 MG1655. For other genomes, the DoriC database (16) provides the sites of replication initiation. If no termination information is available, the site is estimated to be near the *dif* site, or on the opposite side of the circular genome. Replication does not necessarily terminate at the *dif* sites, but it provides a good approximation (17). Origin and termini can also be entered manually when specifying a new genome by selecting ‘other’ in the ‘Choose an organism’ menu.

**Table 1.** Overview of origin of replication and terminus region for strains where methods for recombination with single-stranded oligos have been developed

Organism	Origin of replication	Terminus region	Genbank accession nr.	Sources
*Escherichia coli* str. K-12 substr. MG1655	3923767..3923998	1339769..1682272	NC_000913	ssOligo method: (5) oriC: annotated in GenBank Termini: (12)
*Salmonella typhimurium* LT2	4083788..4084165	1629676..1629703	NC_003197	ssOligo method: (13) oriC: (16) Termini:^a^
*Pseudomonas syringae* str. DC3000	1..338	3211773..3211800	NC_004578	ssOligo method: (13) oriC: (16) Termini:^a^
*Lactobacillus plantarum* WCFS1	1369..1545	1655121..1655148	NC_004567	ssOligo method: (14) oriC: (16) Termini:^a^
*Lactobacillus reuteri* F275	1324..1498	1021118	NC_010609	ssOligo method: (14) oriC: (16) Termini:^b^
*Pantoea ananatis* AJ13355	3803155..3803586	1523700..1523727	NC_017531	ssOligo method: (15) oriC: (16) Termini:^a^

^a^Estimated as being near the *dif* site.

^b^Estimated as being near the opposite side of the genome.

**Figure 2.**
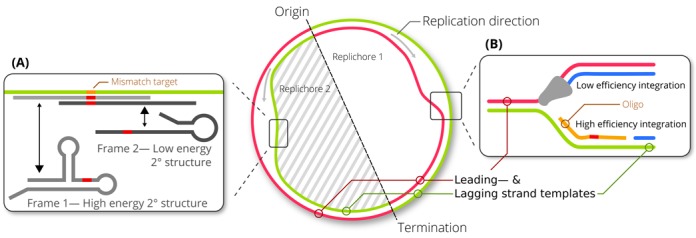
Optimization of oligos to increase recombination efficiency. (**A**) Optimization of the secondary structure of the oligo to avoid high energy secondary structures in the oligo (frame 1). The frame of the oligo is changed to achieve the lowest energy secondary structure (frame 2) with the highest minimum free energy (mfe) possible, within the limitation that the mutation has to be at least 15 bp from the end of the oligo. (**B**) The highest efficiency is achieved when targeting the lagging strand. Therefore, oligos that target the leading strand are reverse complemented.

Another important parameter for oligo optimization is to avoid the formation of stable and closed secondary DNA structures, which will negatively affect the recombineering efficiency (5). MODEST predicts DNA folding by calculating the minimum free energy of the secondary structure of the oligo by employing the *fold* program from the ViennaRNA package (18). The oligo frame is shifted along the genome to find the configuration with the highest minimum free energy. A total of 15 bp of homology is retained at each end of the oligo to prevent decreased integration efficiency because of DNA processing (Figure 2) (19). If desired, optimization of secondary structure can be disabled in the advanced settings, which will result in oligos where mutations are centered.

### MASC primers

MASC PCR is a technique for rapidly assessing the presence of a specific allele (5). For each analyzed locus, three primers are designed: two different forward primers, one specific for the wild-type sequence (fw_wt_) and one specific to the mutated sequence (fw_mut_), and one universal reverse primer. By determining which forward primer leads to the generation of a PCR product in colony PCRs, mutant and wild-type colonies can easily be identified.

MODEST creates MASC primers based on the designed mutations. The algorithm for design of the MASC primers starts at the 3′-end of the wild-type and mutated sequence. From this position, nucleotides are added in the upstream direction until a melting temperature of 62°C (5) of the primer is reached. The melting temperature is calculated with the method described in (20). The fw_mut_ and fw_wt_ primers are then checked to ensure that they are not identical. If they are, the primer design frame is shifted one nucleotide downstream and the process is repeated. This ensures that the two primers generated by the algorithm will only hybridize with either the wild-type or mutant sequence.

The output is in the form of a table with a reference to each oligo via an ID, forward primers for the wild-type and mutant, and a list of reverse primers producing 10 PCR amplicons of different length (100, 150, 200, 250, 300, 400, 500, 600, 700 and 850 bp, respectively). This allows the user to select primer pairs to produce amplicons of a desired size, which facilitates mixing of multiple primer sets that produce different size amplicons in the same MASC PCR reaction.

### Designing oligos for translational gene knockouts

Gene knockouts are traditionally created by complete deletion of the coding sequence. This option is available in MODEST by choosing the delete function. However, replacement or deletion of entire genes is known to have low efficiencies using genome engineering without selectable markers (5). To address the problem with low recombineering efficiencies when introducing large genetic modifications, we developed an alternative option to create translational gene knockouts that combine high recombineering efficiency with the phenotypic stability of a deletion mutant (1). The translational gene knockout operation designs oligos that will introduce tandem stop codons in the first half of the gene sequence. By introducing tandem stop codons, the probability of restoring gene function by acquisition of reverse mutations is drastically reduced because all stop codons need to be simultaneously reverted. The tandem stop codons are also designed to include all three types of stop codons to abolish the possibility of restoring gene function via the acquisition of tRNA suppressor mutations (21). By localizing the mutations to the first half of the gene, the likelihood of producing a truncated protein with some residual activity is minimized.

**Figure 3.**
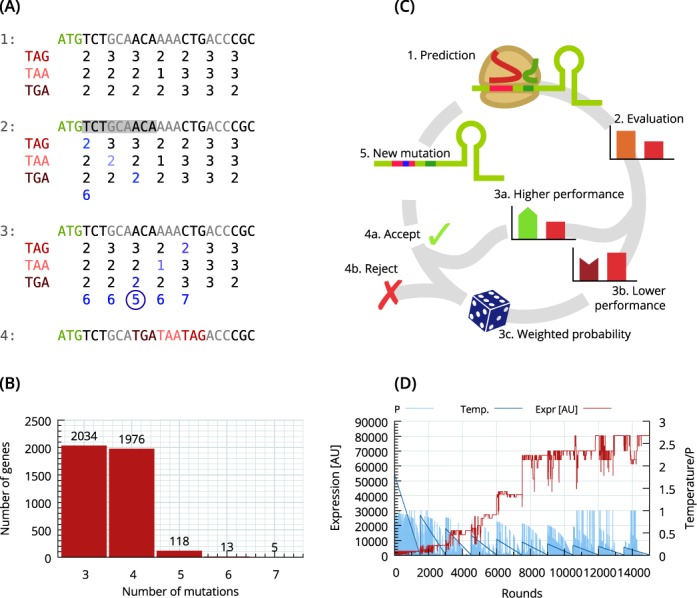
Changes in gene expression by gene knockouts and mutations in the ribosomal binding site. (**A**) Translational knockout module to predict effective knockouts with the fewest number of mutations. The translational gene knockout algorithm works as follows. (1) Calculate matrix of how many mutations are required by each codon to mutate into each stop codon. (2) Calculate the combination containing each stop codon with the least amount of mutations for the chosen window. (3) Slide window along the sequence and calculate best combination for each codon position. (4) Choose a codon position with the least number of mutations and mutate sequence (green: start codon, shades of red: stop codons). (**B**) The theoretical number of mutations required to create translational knockouts by producing three consecutive different stop codons in the first half of the gene was calculated for all genes in *E. coli*. Essential genes cannot be knocked out by this approach. (**C**) Overview of the Monte Carlo simulation that alters the ribosomal binding site sequence through random mutations over several iterations: (1) prediction of expression level for new sequence based on an RBS calculator (8); (2) evaluation of predicted expression level compared to previously evaluated sequence; (3a) sequences with higher performance are always accepted; (4a) sequences with a lower expression are accepted based on a weighted probability; (4b) some are rejected based on a weighted probability. (**D**) A visual overview of the multiple Monte Carlo passes used to target the highest possible predicted expression level. The red line shows the translational expression level in arbitrary units (AU), the dark blue line shows the virtual temperature parameter, and the light blue bars are the probability that a new mutation with a lower predicted expression is accepted.

The default values are three different stop codons in the first half of the gene. To achieve the highest recombineering frequency, a brute-force scoring scheme is used, where every possibility of each stop codon for every position is calculated (Figure 3A). A matrix of how many mutations are required by each codon to mutate into each stop codon is calculated, and a window with the desired number of consecutive stop codons (e.g. three) is then slid along the sequence to calculate the number of mutations required for each window (set of codons). The set of codons that require the fewest mutations are then selected, and if more are present with the same score, the one closest to the start of the gene is preferred. We used the algorithm to design knockout oligos for all genes in *E. coli.* Interestingly, most non-essential genes in *E. coli* are predicted to be efficiently knocked out by this approach with as little as three or four nucleotide substitutions (Figure 3B). The expected replacement efficiency for introducing any gene knockout in a non-essential gene can be calculated to be between 11.6% (seven mutations) and 19.8% (three mutations) (5). Using this method, as few as 5–10 colonies will have to be screened after MAGE to identify a successful knockout, without using any selection marker. Essential genes will be knocked out with greatly reduced efficiency, and only occurs in cells that contain a naturally occurring duplication of the region of interest (22). However, we used all genes in the calculation to not exclude any genes that may be essential only in specific environments (23,24).

### Creating oligos that modulate the translation rate of a target gene

Modulation of expression level to increase the flux to a given metabolite is a key goal in metabolic engineering, but most often only a few predicted targeted modifications can be tested because of practical limits. The sequence of the 5′ untranslated region (5′ UTR), including the ribosome binding site, greatly influences the expression level of a gene (25–28). Wang *et al.* have shown that modulating protein expression with degenerate MAGE oligos, targeting the Ribosome Binding Site (RBS) of 20 different genes, resulted in radically improved lycopene production (1). However, by this approach, most of the applied oligos are redundant, i.e. they achieve similarly low expression levels. This reduces the phenotypic space that can be explored since only a small fraction of the applied oligos will be functional with respect to modulating protein expression levels. High-quality cell libraries with many combinations of genomic changes can be constructed by predicting and introducing precise changes estimated to result in desired levels of expression.

Salis *et al.* have developed an algorithm for predicting protein expression based on thermodynamic calculations of the 5′ UTR and gene sequence (8). We used this prediction algorithm as a basis for identifying gene sequences resulting in desired expression levels and that could be obtained with as few nucleotide substitutions as possible. Because the total number of possible gene sequences that may result in changed expression is too large for calculations within practical time limits, we developed a Monte Carlo simulated annealing method that alters the ribosomal binding site sequence through random mutations over several iterations. For each round, a random position is mutated and the expression of the mutant is scored by calculating the Gibbs free energy change for translation initiation with the algorithm developed by Salis *et al.* (8). The new mutant is selected based on performance and random chance via the standard Metropolis criterion. A mutation is considered better performing when the predicted expression is closer to target expression than the current best candidate. This mutation is accepted as the new best candidate, and all future mutations are based on the best candidate. If the new mutant performs worse than the original best candidate, an acceptance probability is calculated on the basis of the difference between original and new mutant and a ‘virtual temperature’ parameter. The new mutation is randomly accepted as the new best with the calculated probability or rejected, and the original mutation is kept as the best candidate. The simulation starts in a ‘hot’ phase where the temperature parameter is high, which means that the probability of an acceptance is high, and then gradually cools down until the likelihood of acceptance is based solely on performance. The hot-phase step is used to avoid local minima.

The temperature and probability is visualized in Figure 3D, which shows the increasing expression level as the simulation proceeds. As the temperature drops during a pass, the average probability decreases alongside. The whole simulation is implemented in a number of passes, each with several rounds. In the first pass, only one mutation is allowed. If this fails to reach the defined target expression level, an additional mutation is allowed using the previous mutation as the starting point. Another mutation is then allowed until the target expression level or a user-specified limitation is reached (e.g. the parameter max_mutations = 5 limits the number of mutated bases to 5). The beginning of a new pass can be seen in Figure 3D each time the temperature jumps to a new starting temperature. The optimal starting temperature for a number of mutations is automatically estimated.

In case of the 5′ UTR of the targeted gene overlapping the coding sequence of an upstream gene, the RBS module is restricted to only allow wobble sequences that are automatically detected to prevent undesirable amino acid substitutions in the upstream gene.

Users can generate RBS libraries, consisting of several different oligos with different target expression levels, by specifying a parameter (e.g. *n* = 10) to the operation. To achieve a broad range of expression levels, the algorithm aims to collect sequences where the predicted expression level fits an exponential distribution. The operation outputs a list of mutation objects, which is processed by the oligo creation routine and output as optimized MAGE oligos.

### Barcoding of oligos for MO-MAGE

Microarray oligonucleotide MAGE (MO-MAGE) is a novel method to amplify and process oligos from microarray chips for direct use in MAGE to simultaneously perturb thousands of genomic sites (29). Barcoding refers to addition of 20 bp sequences at each end of an oligo suitable for performing PCR. Specific subpools of oligos can be barcoded with unique sequences to allow selective amplification of a subpool from a complex pool of oligos (e.g. several thousands) synthesized on microarrays. The oligos are subsequently enzymatically processed to remove the barcodes. MODEST supports MO-MAGE by allowing the addition of barcodes to individually designed oligos. Oligos can be barcoded by providing a unique barcoding ID when specifying the oligo design. The barcoding ID refers to specific sets of sequences that will be added to each end of the designed oligos. The default barcoding sequences are based on 20 primer sets optimized to avoid cross priming to other barcodes or the *E. coli* genome (30). It is also possible to provide a custom barcoding table in the advanced options by pasting or uploading a text file.

## EXAMPLE

To illustrate the use of MODEST, we have created a simple example where the user desires to create several clones with various levels of increased expression of *lacZ* in the genome encoding beta-galactosidase. The user wants to design a library of 10 oligos that increases expression level of *lacZ* to different levels, one knockout and five oligos downregulating *lacI* (encoding a repressor of *lacZ*) and four oligos upregulating as well as four oligos downregulating *crp* (encoding cAMP receptor protein, which activates transcription of *lacZ*). The goal is to obtain a library of clones with different expression levels of *lacZ*.

The user supplies a project name and specifies the appropriate genome either by using a genome already on the server, by uploading a GenBank file, or supplying a GenBank identifier. When an external genome is used, the user must also specify the origin and terminus of replication or supply an already generated corresponding genome configuration file. The user then selects the desired target (specific gene or the genome) and desired alteration, resulting in automatic generation of an input file. The generated input file can be manually edited and downloaded for reference or later use. Advanced options can be used to specify non-default oligo length or provide barcoding sequences, if desired. In this example, the default options are used (90 bp oligos, no barcoding).

**Figure 4.**
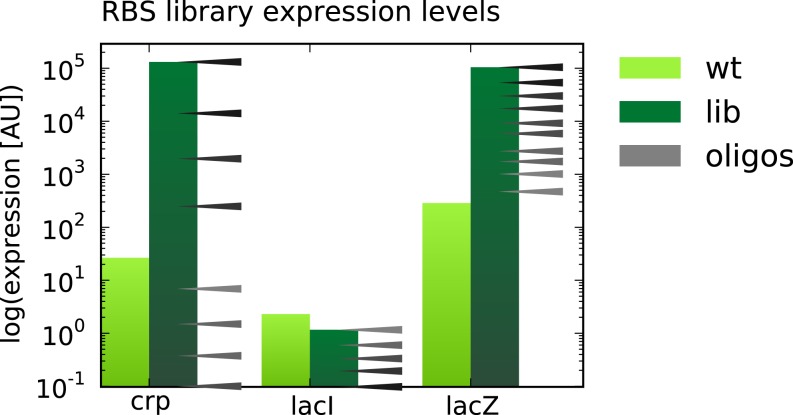
Example of MODEST output, showing a visualization of the *insilico* calculated translation rates expected to result from the mutations in the designed oligos. The light green bar is the predicted wild-type expression level, and the dark green bar is the maximum achieved expression level. The black/gray arrows denote the expression level of the designed oligos.

When the user clicks ‘submit’, the job is started, and a log is displayed and dynamically updated for the user to follow the progression of the job. When the job is completed, the results page appears, stating that the job was successfully completed or any warnings or errors. The designed oligos are shown in a table and can be easily copied to the ordering form format of most commercial suppliers. The user can also access the MASC PCR primers and choose primer sets corresponding to a desired fragment length. All files including input and configuration files as well as output files in a spreadsheet format can be downloaded as a zip file. Oligos designed to change the expression level of a gene are visualized as shown in Figure 4 to provide a graphical overview of the results.

## CONCLUSION

MODEST provides a platform for automatic design of oligos for MAGE and recombineering and is designed for the ease of use for researchers without programming experience. The web server is freely available at http://modest.biosustain.dtu.dk and offers the possibility for automatic oligo design based on user specification of a target genome, a list of desired genomic modifications, or desired phenotypic result such as modified gene expression or gene knockouts. The server will be useful for simpler projects employing recombineering with a few oligos and complex projects involving hundreds of oligos or even thousands with the built-in support of MO-MAGE (29). The tool significantly reduces the time needed for genome engineering projects and enables broader implementation of MAGE by lowering the barrier to get started with a new technique. MODEST will also provide a framework for more advanced projects where oligo design is impossible to perform manually.
